# Evaluation of the efficacy and safety of artemether-lumefantrine in the treatment of acute uncomplicated *Plasmodium falciparum *malaria in Nigerian infants and children

**DOI:** 10.1186/1475-2875-7-246

**Published:** 2008-11-27

**Authors:** Catherine O Falade, Oluwatoyin O Ogunkunle, Hannah O Dada-Adegbola, Adegoke G Falade, Patricia Ibarra de Palacios, Philip Hunt, Mailis Virtanen, Ayoade M Oduola, Lateef A Salako

**Affiliations:** 1 Department of Pharmacology and Therapeutics, University of Ibadan, Nigeria; 2 Institute for Advanced Medical Research and Training, College of Medicine, University College Hospital, Ibadan, Nigeria; 3 Department of Pediatrics, University College Hospital, Ibadan, Nigeria; 4 Department of Medical Microbiology, University College Hospital, Ibadan, Nigeria; 5 Novartis Pharma AG, Basel, Switzerland; 6 Novartis Horsham Research Centre, Horsham, UK

## Abstract

**Background:**

The six-dose regimen of artemether-lumefantrine (AL) is now considered the gold standard for the treatment of uncomplicated *Plasmodium falciparum *malaria. There are few reports evaluating co-artemether in very young Nigerian infants and children. Results of the evaluation of the six-dose regimen in very young infants and children in Nigeria are presented in this report.

**Methods:**

As part of a larger African study, this open label, non-comparative trial, assessed the efficacy and safety of six-dose regimen of AL tablets in 103 Nigerian infants and children weighing between five and 25 kg suffering from acute uncomplicated malaria. Treatment was administered under supervision over three days with children as in-patients. 12-lead ECG tracings were taken pre-treatment and at day 3.

**Results:**

Ninety-three infants and children completed the study as stipulated by the protocol. Mean fever and parasite clearance times for the intent to treat population (ITT) were 24.9 h ± (1.28) and 26 h ± (4.14) and the corresponding figures for the per-protocol population (PP) were 19.24 h ± 13.9 and 25.62 h ± 11.25 respectively. Day 14 cure rates for the ITT and PP were 95.1% and 100% respectively while day 28 cure rates were 91.3% and 95.7% respectively. The overall PCR corrected day 28 cure rate was 95.1% for the ITT. The six-dose regimen of AL was well tolerated with no drug-related serious adverse events. Although six patients recorded a QTc prolongation of > 60 ms on D3 over D0 recording, no patient recorded a QTc interval > 500 ms.

**Conclusion:**

The six-dose regimen of AL tablets is safe and effective for the treatment of acute uncomplicated malaria in Nigerian infants and children weighing between five and 25 kg.

**Trial registration:**

NCT00709969

## Background

The deleterious consequences of malaria continue to increase in endemic areas as a result of the emergence and widespread dissemination of drug resistant *Plasmodium falciparum *[1,2]. Children below five years of age are most at risk in endemic countries. Safe and effective drugs with long effective clinical lives for malaria therapy are urgently needed in Africa. Artemether-lumefantrine (AL) is a fixed-dose combination tablet containing 20 mg of artemether and 120 mg of lumefantrine. This combination combines the benefits of a rapid schizonticidal effect of artemether with a slow but longer acting schizonticidal effect of lumefantrine. AL was first made available as a four-dose regimen, which was found to be safe and efficacious in areas where drug resistance in *P. falciparum *was rare at the time, such as China and India [3,4], but not in Thailand, an area where multi-drug resistance is common [5]. Subsequent trials of the six-dose regimen in Thailand found it to be well tolerated with high cure rates similar to those with high dose mefloquine plus artesunate – the standard of care for acute uncomplicated malaria in Thailand [6,7]. The six-dose regimen is now the global standard regimen for AL [8].

The safety and efficacy of the six-dose regimen of AL in infants and children weighing between five and 25 kg for the treatment of acute uncomplicated falciparum malaria was evaluated in this study. Special attention was paid to the response to treatment by non-immune infants between five and 10 kg. Infants weighing between five and 10 kg, who had never had an episode of malaria prior to the current episode were considered to be non-immune to malaria. Evaluations for neurotoxicity were included in this study based on reports of neurotoxicity associated with artemisinins [9,10]. In addition, electrocardiographic recordings were performed on days 0 and 3, due to the structural similarity between lumefantrine and halofantrine, although previous studies had unequivocally demonstrated that lumefantrine lacks the cardiotoxicity of halofantrine [7]. Cardiotoxicity (QTc interval prolongation and arrhythmias) associated with the use of the latter compound has been reported [11].

This report is based on data from the "Nigerian Coartem^® ^Treatment Group" of the multicentre study evaluating the efficacy and safety of AL in the management of malaria in African infants and children [12].

### Patients and methods

The study was conducted at the General Out Patient Clinic and paediatric wards of the University College Hospital, Ibadan, south-western Nigeria between August 2002 and February 2003. Malaria transmission is intense throughout the year in the study area with peak transmission occurring during the rainy season months of May to October. Ethical approval for the study was obtained from the Joint University of Ibadan/University College Hospital Institutional Review Committee and the ethical review board of the World Health Organization, Geneva, before commencement. The study was conducted according to the declaration of Helsinki and its amendments. Good clinical practice was also adhered to and a written informed consent or witnessed verbal informed consent was obtained from the parent or guardian of each enrolled child before any study related procedure was performed.

### Study design

In an open label, non-comparative study design, children weighing between five and 25 kg suffering from confirmed acute uncomplicated falciparum malaria were treated with AL (Coartem^®^). An open label study design was chosen because at the time there were no affordable products approved by regulatory authorities for the treatment of malaria in children weighing < 10 kg; thus no suitable comparator was available. The study population was stratified into three body weight groups as follows: 5-<10 kg, 10-<15 kg and 15-≤25 kg as body weight groups (BWG) 1, 2 and 3 respectively. Children weighing 5-<10 kg (BWG1) constituted half of the study population in the design so as to have enough data for evaluation in this group of children.

Inclusion criteria for the study were body weight 5 kg to < 25 kg, microscopically confirmed symptomatic acute uncomplicated falciparum malaria, asexual parasite density between 1,000 and 100,000/μL, axillary temperature ≥ 37.5°C and informed consent by the parent or guardian. Patients who had clinical features of severe malaria, other plasmodial infection, severe malnutrition, history of having received halofantrine or any other drug known to influence cardiac function within four weeks prior to the screening visit were excluded from the study. Other exclusion criteria were severe gastrointestinal disease or hypersensitivity to the study drugs. Patients were withdrawn from the study if a serious adverse event, abnormal laboratory values or test procedures, unsatisfactory therapeutic effect or protocol violation occurred. Reasons for discontinuation from the study were withdrawal of consent, vomiting of replacement dose of study drug, loss to follow-up or death.

### Clinical and laboratory evaluation

Patients fulfilling eligibility criteria were allocated to the appropriate body weight group. A complete medical history, physical examination including vital signs, specific neurological examination and baseline signs and symptoms of malaria for specific safety evaluation of treatment emergent signs and symptoms (TESS) was carried out. All worsening, and newly occurring signs and symptoms of malaria after baseline, whether considered drug-related or not, but before recurrence of parasitaemia were defined as TESS. Thick and thin blood films were prepared from a finger prick and stained with 10% freshly prepared Giemsa stain and examined using x1,000 magnification of a light microscope for parasite detection, quantification and morphology. Parasite density was calculated using individual patient's white blood cell count. Blood (3.5 ml) was collected for haematology and clinical chemistry evaluation on days 0, 3 and 28. Haematology evaluation included haematocrit, haemoglobin, red blood cell count, white blood cell count with differential, platelet count and glucose-6-phosphate dehydrogenase activity, while blood chemistry evaluation included blood glucose, serum bilirubin, creatinine, alanine transaminase (ALT), aspartate transaminase (AST) and γ-glutamyl transferase. Urine measurements were performed when considered necessary and parameters evaluated included specific gravity, haemoglobinuria, proteinuria and sediment. Blood spots were blotted on isocode stix for polymerase chain reaction (PCR) before commencement of drug therapy and at recurrence of parasitaemia for genotyping of isolates to distinguish between recrudescence and re-infection [13]. In addition, 12-lead electrocardiographic tracings were recorded before treatment and at day 3. Haematology, blood chemistry and urine examinations were repeated at days 3 and 28. Therapeutic response was assessed by clinical and parasitological parameters.

### Drug therapy and follow-up

The study consisted of two phases: a treatment phase and a post treatment phase. During the first four days, termed the treatment phase, patients were admitted to the ward for drug therapy. Enrolled patients received Coartem^® ^(Novartis Pharma AG; Basel) which consists of artemether (20 mg) and lumefantrine (120 mg) per tablet supervised by a doctor or nurse. Children weighing 5 to < 15 kg received 6 doses of 1 tablet each of AL at 0, 8, 24, 36, 48 and 60 hours while children weighing 15 to < 25 kg received 2 tablets of study drug at similar time points as above. Study drug was administered with sweetened condensed milk for older children and feeding encouraged soon after dosing while younger children took the study drug with water and were breast-fed immediately following drug administration. Patients were subsequently seen as outpatients daily for four days (up to day 7) and then on days 14, 21 and 28 during the post treatment phase.

### Efficacy assessment

Efficacy evaluation was based on parasitological cure rates at days 7, 14 and 28. Cure rates at days 7, 14 and 28 were defined as the proportion of patients cleared of asexual parasitaemia within the specific time intervals of initiation of treatment with AL without recrudescence within the specified days. Re-infection was distinguished from true recrudescence by genotyping using PCR [13]. In addition to cure rates, mean fever clearance time (FCT) and mean parasite clearance time (PCT) were determined. PCT was defined as the time elapsing from drug administration until the clearance of parasitaemia. FCT was defined as the time from drug administration until axillary temperature fell below 37.5°C and remained so for at least 48 hours. All patients with recurrence of parasitaemia received oral amodiaquine (25 mg/kg body weight) over 3 days plus oral sulphadoxine-pyrimethamine (25 mg/kg of sulphadoxine component to the nearest next quarter tablet) as a single dose co-administered on the day of starting re-treatment.

### Safety evaluation

Safety evaluation was based on the intent to treat population (ITT). Safety assessment consisted of monitoring and recording of all adverse events whether volunteered, discovered by questioning or detected by examination in addition to clinically significant changes in haematology, blood chemistry and urine values. Adverse events information was collected by evaluating TESS. An adverse event was regarded as any undesirable sign, symptom or medical condition occurring after starting the study drug, whether the event was considered to be related to study drug or not. Any signs and symptoms that appeared newly or worsened from baseline were recorded as adverse events. Standard 12-lead electrocardiographic tracings were done pre-treatment on day 0 and at day 3 as part of safety evaluation. Primary data as well as changes from baseline were derived from an independent blinded review of electrocardiographic results. Corrected QTc interval values were calculated according to Bazett's formula (QTc = QT/vRR) and Fridericia's formula (QTc/_3_vRRms). Values < 430 and 450 ms were classified as normal for males and females respectively, 431–450 and 451–470 ms, borderline while > 450 and > 470 ms were classified as prolonged.

### Statistical methods

Safety variables were summarized descriptively by body weight group and overall. ITT and safety populations were defined as all patients who received at least one dose of study medication. Adverse events, including signs and symptoms of malaria that had increased in intensity as compared to baseline, were summarized by MedDRA primary system organ class, preferred term, intensity, and relationship to study drug. Laboratory data, including changes from baseline, were summarized with descriptive measures by time point and classifications according to the reference ranges were summarized in shift tables. ECG data were summarized accordingly. All efficacy analysis was based on the ITT with supportive analyses on the per-protocol population (PP). The PP population was made up of ITT patients who received all six doses of medication and who adhered to the study protocol. The variables were summarized using descriptive statistics (mean, standard deviation, median, minimum and maximum or frequency and percentage, as appropriate) by body weight group and overall. PCT and FCT were derived by means of the Kaplan-Meier method. 95% confidence intervals for the cure rates were determined using the exact Pearson-Clopper limits. Time to fever clearance and time to parasite clearance were evaluated using Cox's proportional hazards regression analysis.

## Results

### Patient demographics at presentation

One hundred and three consecutive children screened between August 2002 and February 2003 at the study centre and who fulfilled the inclusion criteria were enrolled in the study. The distribution of the ITT population consisted of 50 in BWG 1, 37 in BWG 2 and 16 in BWG 3 (Table 1). The youngest child in this study was five weeks old. The age distribution of children enrolled in the study is shown in Table 2. There were 50 males and 53 females in the ITT population. Safety evaluation was based on the ITT population which is also the safety population. Ninety-three of 103 (90.3%) enrolled patients completed the study as stipulated by the protocol. The PP population consisted of 45 in BWG1, 34 in BWG2 and 14 in BWG3. Gender disposition of PP was similar to that of ITT. Reasons for exclusion of 10 patients from the PP included loss to follow up (2), withdrawal of consent (1) protocol violation (6) and death (1). One patient in BWG2 died on the ninth day of study from severe gastroenteritis, which was not considered drug-related. Criteria for withdrawal for protocol violation in the study included unauthorized use of anti-malarial medication during follow up and more than one parasite count missing. All patients excluded from the PP population except those who were lost to follow up and the child who died were followed up to 28 days for safety evaluation.

**Table 1:** Clinical features at baseline of children suffering from acute uncomplicated malaria by body weight group (ITT population)

**Characteristic**	**BWG1**	**BWG2**	**BWG3**	**TOTAL**
Number enrolled and treated (ITT)	50	37	16	103
Sex (M: F)	21:29	21:16	8:8	50:53
Age (years)				
Mean ± SD	1.3 ± 0.56	3.4 ± 1.3	6.5 ± 1.76	2.8 ± 2.13
Range	0.2 – 3.1	0.9 – 6.8	4.0 – 9.9	0.2 – 9.9
Non-Immune (n)	15	-	-	15
Weight (kg)				
Mean ± SD	8.0 ± 1.05	12.3 ± 1.52	18.7 ± 1.87	11.2 ± 4.0
Range	5.1 – 9.6	10 – 14.5	15.5 – 22	5.1 – 22
Parasite density (/μL)				
Mean ± SD	21,286 ± 19,900	32,668 ± 27,400	27,656 ± 26,074	26,364 ± 24,135
Range	2,927–95,546	2,112–106,425	2,332–104,919	2,112–106,426
Temperature (°C)				
Mean ± SD	38.9 ± 0.80	38.8 ± 0.86	39.0 ± 0.64	38.9 ± 0.80
Range	37.6 – 40.8	37.6 – 40.3	37.9 – 39.8	37.6 – 40.8
Haemoglobin (g/L)				
Mean ± SD	90.2 ± 14.73	92.7 ± 16.41	103.4 ± 15.87	93.3 ± 16.04
Haematocrit (%)				
Mean ± SD	27.9 ± 4.12	28.6 ± 4.17	30.9 ± 4.77	28.6 ± 4.33

**Table 2:** Age distribution of patients at baseline by body weight group

**Age group (Months)**	n (%)			
	
	**BWG1** **5-<10 kg** **N = 50**	**BWG2** **10-<15 kg** **N = 37**	**BWG3** **15-<25 kg** **N = 16**	**Total** **5-<25 kg** **N = 103**
0- = 6	3 (6)	----	----	3 (2.9)
> 6 – 12	17 (34)	1 (2.7)	----	18 (17.5)
> 12 – 24	26 (52)	3 (8.1)	-----	29 (28.2)
> 24 – 48	4 (8)	24 (64.9)	1 (6.3)	29 (28.2)
> 48 – 72	---	8 (21.6)	7 (43.8)	15 (14.6)
> 72	---	1 (2.7)	8 (50)	9 (8.7)
TOTAL	50 (100)	37 (100)	16 (100)	103 (100)

### Efficacy assessment

*P. falciparum *was the only species identified in the blood films of enrolled patients. Baseline parasite density was similar in all body weight groups (Table 1). A total of 75.3% and 89.9% of patients were free of patent parasitaemia at 24 and 60 hours, respectively, while all patients were free of patent parasitaemia by day 3 evaluation. Cure rates in PP at day 7 and day 14 were 100% for all body weight groups while the cure rates in the ITT at the same time points were 98.1% and 95.1%. Day 28 cure rates in the PP population without PCR correction were 95.6%, 94.1% and 100% among children in BWG1, 2 and 3 respectively while day 28 cure rates for BWG1, 2 and 3 among the ITT populations were 92%, 89.2% and 93.8%. These gave overall uncorrected cure rates of 95.7% and 91.3% for the PP and ITT populations on day 28 respectively. Four patients had patent parasitaemia at day 28; all four were shown to be new infections by PCR. Details of response of infection to therapy are shown in Table 3. In addition, fever clearance in response to AL was rapid. 95.6%, 97.1%, 100% of children in BWG1, 2 and 3 respectively, recorded axillary temperatures below 37.5°C by 36 hours after starting drug treatment.

**Table 3:** Clinical and parasitological responses to treatment with artemether-lumefantrine by body weight group

	**Body weight group**			
**Characteristic**	**BWG1** **5-<10 kg**	**BWG2** **10-<15 kg**	**BWG3** **15-<25 kg**	**Total** **5-<25 kg**
Cure rates ITT %(n/M)^1 ^(uncorrected)	N = 50	N = 37	N = 16	N = 103
Day 7	98.0% (49/50)	97.3% (36/37)	100.0% (16/16)	98.1% (101/103)
Day 14	96.0% (48/50)	94.6% (35/37)	93.8% (15/16)	95.1% (98/103)
Day 28	92.0% (46/50)	89.2% (33/37)	93.8% (15/16)	91.3% (94/103)
*95% CI*	(80.8–97.8)	(74.6–97.0)	(69.8–99.8)	(84.1–95.9)
				
Day 28 (PCR corrected)	96.0% (48/50)	94.6% (35/37)	93.8% (15/16)	95.1% (98/103)
*95% CI*	(86.3–99.5)	(81.8–99.3)	(69.8–99.8)	(89.0–98.4)
				
Cure rate PP %(n/M)^1 ^(uncorrected)	N = 45	N = 34	N = 14	N = 93
Day 7	100% (45/45)	100% (34/34)	100% (14/14)	100% (93/93)
Day 14	100% (45/45)	100% (34/34)	100% (13/13)	100% (92/92)
Day 28	95.6% (43/45)	94.1% (32/34)	100% (13/13)	95.7% (88/92)
*95% CI*	(84.9–99.5)	(80.3 – 99.3)	(-)	(89.2 – 98.8)
				
PCT (h) ITT	N = 50	N = 37	N = 16	N = 103
Mean ± SE	24.8 ± 1.3	26.2 ± 2.31	21.2 ± 4.33	24.9 ± 1.28
Range	5.3 – 47.8	7.7 – 59.9	7.2 – 71.1	5.3–71.1
Median	23.8	23.8	23.0	23.8
95%CI^2^	(23.7–23.9)	(23.1–35.3)	(7.9–23.8)	(23.7–23.8)
FCT (h) ITT)	N = 50	N = 37	N = 16	N = 103
Mean ± SE	24.5 ± 3.81	17.6 ± 2.04	44.2 ± 19.98	26.1 ± 4.14
Range	3.5 – 163.8	4.1 – 47.5	7.2–308.7	3.5–308.7
Median	23.4	8.2	15.4	22.4
(95% CI)^2^	(7.8–23.8)	(7.8–23.4)	(7.8–34.6)	(7.9–23.5)

^1^In some instances N is different from M as M = number of patients who had the measurement at given time point

^2^The CI is provided for the median

### Safety assessment

The study drug was well tolerated and no patient was withdrawn due to recurrent vomiting or poor tolerability. Anaemia (n = 59) was the most frequently reported adverse event by laboratory evaluation, while cough (n = 33) was the most often volunteered adverse event reported. Respiratory infection (n = 31), diarrhoea (n = 20), vomiting (n = 18), rash (n = 17), clonus (n = 13) and insomnia (n = 11) were the next six most common treatment emergent adverse events recorded after baseline but before recurrence of parasitaemia among study participants. Other adverse events recorded among patients included hypothermia (n = 6), hyperreflexia (n = 6) and distended abdomen (n = 1). Five of the six episodes of hypothermia occurred among children in BWG1. Mean haemoglobin level decreased from 93.3 ± 16.04 g/L on day 0 to 83.2 ± 13.43 g/L on day 3 and increased to 103.2 ± 14.76 g/L on day 28 while the number of children with low neutrophil count on day 0 decreased from 87 to 40 at day 28. In addition there was no record of eosinophilia, intravascular haemolysis or leucopaenia (total white blood cell count < 2,500/mm^3^) during the study. Biochemical parameters were not adversely affected by the study drug.

### Serious adverse events

One serious adverse event was recorded during the study. A child in BWG2 died at home on day 9 of the study within a few hours of onset of severe gastroenteritis without having been seen by the study team or by any medical personnel. This serious adverse event was not considered drug-related. The study team received information of the death a day after the event when two other siblings (who were not enrolled in the study) developed similar illnesses and were brought to the study team for treatment. The siblings were diagnosed as having cholera. They responded well to treatment and went home well recovered. A verbal autopsy was conducted to identify the cause of death of the study participant.

### Electrocardiographic parameters

No patient reported any symptomatic cardiac adverse event. Mean heart rate and RR intervals at enrollment were 140.8 ± 21.41/min and 437.0 ± 70.15 ms while day 3 figures were 119.8 ± 21.36/min and 518.4 ± 100.8 ms respectively. There was a slight increase in mean PR and QTc intervals between day 0 and day 3. The mean PR interval increased from 117.7 ± 14.84 ms at enrollment to 126.9 ± 19.52 ms on day 3. The mean QTc values on days 0 and 3 were 400.6 ± 23.64 ms and 410 ± 21.31 ms respectively. There was no record of a QTc interval greater than 500 ms using Bazett's formula. However 6 patients (3, 2, & 1 in BWG1, BWG2 and BWG3 respectively) recorded QTc interval prolongation of over 60 ms on day 3 when compared with day 0. All were asymptomatic.

## Discussion

This report presents the details of data obtained in the Nigerian arm of the multicentre study evaluating the safety and efficacy of AL in Kenya, Tanzania and Nigeria [12]. The six-dose regimen of AL was found to be well tolerated, safe and highly efficacious in the treatment of acute uncomplicated *P. falciparum *malaria in Nigerian children and infants weighing as little as 5 kg. The youngest child in this study was five weeks of age. Three of the enrollees were less than six months of age while 48.5% were less than two years of age (Table 2).

The main thrust of the multicentre study reported by Falade *et al *[12], is the safety of the six-dose regimen of AL. This paper presents a more detailed report of the findings among Nigerian children with a view to providing additional information to practitioners in Nigeria who are often anxious about the choice of anti-malarial drugs outside chloroquine and sulphadoxine-pyrimethamine when treating very young infants. The good safety profile obtained among Nigerian children treated with AL is comparable with findings during pre-registration studies of the four-dose regimen in Nigeria [14-17]. There were no reports of drug-related serious adverse events. In the multicentre study [12], only one (urticaria) of six serious adverse events was considered drug-related. Other recorded adverse events during the study were generally mild. Adverse events such as anaemia, cough, diarrhoea and vomiting (Tables 4 and 5) are difficult to distinguish from the clinical features of the disease (malaria) being treated. Tolerability of the drug was also quite good and no patient was withdrawn because of recurrent vomiting. There were 17 episodes of rash among the Nigerian children enrolled in the study. The rashes were sometimes pruritic, but were all transient clearing spontaneously within three days without any specific therapy. Some (7) of the rashes were classified as possibly drug-related. Lefèvre *et al *[7] had earlier reported similar episodes of pruritic rash in a study in Thailand. Hypothermia was recorded in six children (5 BWG1, 1 BWG2) during routine six-hourly temperature recording – a routine ward practice in the hospital where the study was conducted. There was no mention of hypothermia in the larger study report by Falade *et al *[12] as only adverse events which occurred in > 2% of the total study population were listed. The significance of the hypothermia is uncertain, as over 40% of children in BWG1 were less than 12 months of age (Table 2). The delicate body temperature regulation in this age group is well recognized.

**Table 4:** Adverse events after baseline but before recurrence of parasitaemia occurring in > 5% of patients, by body weight group

**Adverse Events**	**n (%)**			
	
	**BWG1** **5-<10 kg** **(N = 50)**	**BWG2** **10-<15 kg** **(N = 37)**	**BWG3** **15-<25 kg** **(N = 16)**	**Ttotal** **5-<25 kg** **(N = 103)**
Anaemia	32 (64.0)	20 (54.1)	7 (43.8)	59 (57.3)
Cough	20 (40.0)	11 (29.7)	2 (12.5)	33 (32.0)
Hepatomegaly	9 (18.0)	12 (32.4)	5 (31.3)	26 (25.2)
Respiratory tract infection	21 (42.0)	9 (24.3)	1 (6.3)	31 (30.1)
Splenomegaly	10 (20.0)	9 (24.3)	2 (12.5)	21 (20.4)
Diarrhoea	11 (22.0)	7 (18.9)	2 (12.5)	20 (19.4)
Vomiting	10 (20.0)	3 (8.1)	5 (31.3)	18 (17.5)
Rashes	11 (22.0)	5 (13.5)	1 (6.3)	17 (16.5)
Anorexia	9 (18.0)	5 (13.5)	1 (6.3)	15 (14.6)
Clonus	7 (14.0)	5 (13.5)	1 (6.3)	13 (12.6)
Insomnia	7 (14.0)	3 (8.1)	1 (6.3)	11 (10.7)
Catarrh	4 (8.0)	5 (13.5)	0 (0.0)	9 (8.7)
Hypothermia	5 (10.0)	1 (2.7)	0 (0.0)	6 (5.8)
Hyperreflexia	3 (6.0)	2 (5.4)	1 (6.3)	6 (5.8)

**Table 5:** Adverse events (occurring after baseline but before recurrence of parasitaemia in > 2% of children) suspected by investigators to be related to study medication by body weight group

**Adverse Events**	**n (%)**			
	
	**BWG1** **5-<10 kg** **(N = 50)**	**BWG2** **10-<15 kg** **(N = 37)**	**BWG3** **15-<25 kg** **(N = 16)**	**Total** **5-<25 kg** **(N = 103)**
At least one adverse event	30 (60.0)	16 (43.2)	6 (37.5)	52 (50.5)
Anaemia	7 (14.0)	6 (16.2)	1 (6.3)	14 (13.6)
Diarrhoea	5 (10.0)	5 (13.5)	1 (6.3)	11 (10.7)
Clonus	7 (14.0)	5 (13.5)	1 (6.3)	13 (12.6)
Vomiting	7 (14.0)	0 (-)	4 (25.0)	11 (10.7)
Rashes	4 (8.0)	3 (8.1)	0 (-)	7 (6.8)
Hyperreflexia	1 (2.0)	2 (5.4)	1 (6.3)	4 (3.9)
Cough	2 (4.0)	1 (2.7)	0 (-)	3 (2.9)

Earlier reports of neurological abnormalities in some experimental animals notably dogs and Sprague-Dawley rats [9,10] during toxicological studies have been a major concern in the therapeutic use of artemisinin derivatives. Special attention was paid to neurological TESS evaluation pre-enrollment and at days 3, 7, 14 and 28 evaluations. It is noteworthy that no significant neurological abnormalities were recorded during this study. Transient ankle clonus, hyperreflexia and insomnia were the only neurological abnormalities observed among the children enrolled in Nigeria. These observations were made during the treatment phase of the study. Anxiety induced by hospitalisation and associated ward routines (twice daily TESS and blood film preparation, drug administration etc) could explain some of these observed neurological adverse events. The lack of electrocardiographic evidence of cardiotoxicity and specifically on the QTc evaluation during this study is consistent with previous findings in both healthy volunteers and in patients suffering from acute uncomplicated malaria who received AL and confirms previous findings that lumefantrine lacks the cardiotoxicity of halofantrine [18,19].

The PCR corrected cure rates on days 7, 14 and 28 were 100% in all body weight groups. Additional efficacy indicators were rapid fever clearance and parasite clearance (Table 3). Other clinical features associated with malaria apart from fever also cleared rapidly. These results are consistent with previous studies [7,14,20]. Abdu-Aguye *et al *[14] in a pre- registration study of the four-dose regimen in Zaria, Northern Nigeria recorded a 100% cure rate at day 14 among 50 patients aged two to 65 years suffering from acute uncomplicated malaria. On the other hand, the efficacy results recorded in this report are superior to those obtained from some earlier studies with the four-dose regimen in Nigeria. Salako *et al *[17] reported cure rates of 87% and 73% on days 7 and 14 respectively among children aged two to 12 years, Ezedinachi *et al *[16] reported 96% and 93% cure rates on days 7 and 14 from Calabar, Nigeria. Also working in Nigeria, Eke *et al *[15] recorded a day 7 cure rate of 88% among 57 patients aged two to 16 years in Port Harcourt.

The superior efficacy of the six-dose regimen used in this study when compared with that of the four-dose regimen is consistent with findings in similar studies that evaluated the four-dose and six-dose regimens in the western border of Thailand, in Tanzania, Kenya and the Gambia [7,12,21]. Meremikwu *et al *[22] working in Calabar, Nigeria recorded a day 14 uncorrected PCR cure rate of 87% among the 54 children they studied (evaluable population). Two of the children in their study had late clinical failure, while five had late parasitological failure. The PCR uncorrected day 14 cure rate in the study being reported was 100% among the PP population. However, Meremikwu *et al *[22] did not report the PCR corrected cure rate, and so could not differentiate between re-infection and recurrence. A day 14 uncorrected cure rate of 87% is not unexpected in a high transmission geographical region where re-infection is frequent. In addition, there are other factors that could account for this such as a smaller per protocol population of 54 compared to 93 patients in the present study. The patients in this study were admitted during the treatment phase of the study thus ensuring 100% supervised dosing. In addition, AL was administered with a fatty meal: sweetened condensed milk for older children while infants were breast-fed soon after dosing during this study. This was done to enhance the bioavailability of lumefantrine. Ashley *et al *[23], in a recent pharmacokinetic study of healthy volunteers, reported a dose response relationship between the volume of soya milk administered and lumefantrine bioavailability. Furthermore the study reported by Ashley *et al *showed that administration of AL with soya milk at a quantity which corresponds to 1.2 g of fat increased lumefantrine area under the curve (AUC) by more than five fold. This underscores the importance of educating health care workers, patients and caregivers of the importance of compliance and the need to administer AL with food that contains fat, be it conventional milk, soya milk or some appropriate local food.

Artemisinin containing anti-malarial combinations is known to induce rapid parasite clearance and high parasitological cure rates. This expectation was met by the response profile obtained in this study. Approximately 75% of the PP population was free of patent parasitaemia 24 hours after initiation of therapy while none of the study population had detectable peripheral parasitaemia by day 3.

## Conclusion

In conclusion, the six-dose regimen of AL is safe and effective in the management of acute uncomplicated falciparum malaria in Nigerian infants and children weighing as little as five kg, including non-immune children in an area of worsening drug resistant falciparum malaria. The results of this study suggest that if properly deployed, AL could lead to a reduction and may also contribute to a significant extent in halting the worsening morbidity and mortality from malaria on the African continent [1,2]. The Federal government of Nigeria recently changed its malaria treatment policy from chloroquine to artemisinin-based combination therapy as a first line drug with a preference for AL and artesunate plus amodiaquine in that order [24]. It is important to design appropriate information, education and communication materials, which will educate patients, parents, guardians and health care workers alike on correct treatment regimen and the need to administer AL with food and that will encourage compliance as part of the efforts at prolonging the useful therapeutic life of this combination.

## Abbreviations

AL: Artemether-lumefantrine; ALT: Alanine transaminase; AST: Aspartate transaminase; BWG: Body weight group; CI: Confidence interval; FCT: Fever clearance time; IEC: Information, education and communication; ITT: Intent to treat population; PCT: Parasite clearance time; PP: Per protocol population; TESS: Treatment emergent signs and symptoms; /μL: per micro litre;

## Competing interests

COF, OOO, HOD-A, AGF, AMJO and LAS declare no competing interests. PH and MV are employees of Novartis Pharma AG and PidP was an employee of Novartis at the time this work was undertaken.

## Authors' contributions

COF developed the study design, participated in the study conduct and interpretation of the data, drafted and critically reviewed the paper. OOO participated in the study conduct and interpretation of the data, and edited the draft manuscript. HOD-A and AGF participated in the study conduct and edited the draft manuscript. PIdP, PH, MV, AMJO and LAS participated in interpretation of data and critically reviewed the draft manuscript. All authors read and approved the final version of the manuscript.

## References

[B1] Zucker JR, Ruebush, TK II, Obonyo C, Otieno J, Campbell CC (2003). The mortality consequences of chloroquine use in Africa: Experience in Siaya, Western Kenya. Am J Trop Med Hyg.

[B2] Trape JF (2001). The public health impact of chloroquine resistance in Africa. Am J Trop Med Hyg.

[B3] Jiao X, Liu GY, Shao CO, Zhao X, Galliman I, Royce C (1999). Phase II trial in China of a new rapidly acting and effective oral antimalarial CGP 56697 for the treatment of *Plasmodium falciparum *malaria. Southeast Asian J Trop Med Public Health.

[B4] Kshirsagar NA, Gogtay NJ, Moorthy NS, Gard MR, Dalvi SS, Chogle AR, Sorabjee JS, Marathe SN, Tilve GH, Bhatt AD, Sane SP, Mull R, Gathmann I (2000). A randomized, double blind, parallel-group, comparative safety and efficacy trial of oral co-artemether versus oral chloroquine in the treatment of acute uncomplicated *Plasmodium falciparum *malaria in adults in India. Am J Trop Med Hyg.

[B5] van Vugt M, Brockman A, Gemperli B, Luxemburger C, Gathmann I, Royce C, Slight T, Looareesuwan S, White NJ, Nosten F (1998). Randomized comparison of artemether-benflumetol and artesunate-mefloquine in treatment of multidrug resistant falciparum malaria. Antimicrob Agents Chemother.

[B6] van Vugt M, Nilairataria P, Gemperli B, Fathman I, Phaipun L, Broockman PA, Luxemburger C, White NJ, Nosten F, Looareesuwan S (1999). Efficacy of six-doses of artemether-lumefantrine (benflumetol) in multi drug resistant *Plasmodium falciparum *malaria. Am J Trop Med Hyg.

[B7] Lefèvre G, Looareesuwan S, Treeprasertsuk S, Krudsood S, Silachamroon I, Mull R, Bakshi R (2001). A clinical and pharmacokinetic trial of six doses of artemether-lumefantrine for multidrug-resistance *Plasmodium falciparum *malaria in Thailand. Am J Trop Med Hyg.

[B8] Makanga M, Premji Z, Falade C, Karbwang K, Mueller EA, Andriano K, Hunt P, Ibarra de Palacios P (2006). Efficacy and safety of the six-dose regimen of artemether-lumefantrine in pediatrics with uncomplicated *Plasmodium falciparum *malaria: a pooled analysis of individual patient data. Am J Trop Med Hyg.

[B9] Brewer TG, Grate SJ, Peggins JO, Weina PJ, Petras JM, Levine BS, Heifer MH, Schuster BG (1994). Fatal neurotoxicity of artemether and arteether. Am J Trop Med Hyg.

[B10] Kamchonwongpaisan S, McKeever P, Hossler O, Ziffer H, Meshinick SR (1996). Artemisinin neurotoxicity: Neuropathology in rats and mechanistic studies in-vitro. Am J Trop Med Hyg.

[B11] Castot A, Rappaport P, LeCoz P (1993). Prolonged Q-Tc interval with halofantrine. Lancet.

[B12] Falade C, Makanga M, Premji Z, Ortmann C-E, Stockmeyer M, Ibarra de Palacios P (2005). Efficacy and safety of six dose artemether-lumefantrine (Coartem^®^) tablets (six-dose regimen) in African infants and children treatment of acute uncomplicated malaria. Trans R Soc Trop Med Hyg.

[B13] Snounou G, Beck H (1998). The use of PCR genotyping in the assessment of recrudescence or reinfection after antimalarial drug treatment. Parasitol Today.

[B14] Abdu-Aguye I, Gebi UI, Wuyat A, Agbo M (2000). An open-label uncontrolled trial confirming efficacy and safety of the 4-dose regimen of Coartem® in the treatment of acute Plasmodium falciparum malaria in patients aged > 2 years in Zaria Nigeria.

[B15] Eke F, Akhidue V, Ukiwo UE, Akosubo IJ, Izuora P, Akpan PU, Ufford M, Ugwumba F, Dennis A (2000). Report of an open-label uncontrolled trial confirming efficacy and safety of a 4-dose regimen of Coartem® [artemether-lumefantrine (benflumetol)] in the treatment of acute Plasmodium falciparum malaria in children in Port Harcourt.

[B16] Ezedinachi EN, Ekanem-Ephraim E, Ndifon N, Amaechi V, Alaribe AA, Bam AB (2000). An open-label uncontrolled trial confirming efficacy and safety of the 4-dose regimen of Coartem® in the treatment of acute Plasmodium falciparum malaria in patients aged > 2 years in Calabar Nigeria.

[B17] Salako LA, Adewole TA, Afolabi BM, Mafe AG (2000). An open-label uncontrolled trial confirming efficacy and safety of the 4-dose regimen of Coartem® in the treatment of acute Plasmodium falciparum malaria in patients aged > 2 years in Lagos Nigeria.

[B18] van Vugt M, Ezzet F, Nosten F, Gathmann I, Wilairatana P, Looareesuwan S, White NJ (1999). No evidence of cardiotoxicity during treatment with artemether-lumefantrine. Am J Trop Med Hyg.

[B19] Bindschedler M, Lefèvre G, Degen P, Sioufi A (2002). Comparison of the cardiac effects of the antimalarials co-artemether and halofantrine in healthy participants. Am J Trop Med Hyg.

[B20] Hatz C, Abdulla S, Mull R, Schellenberg D, Gathmann I, Kibatala P, Beck H, Tanner M, Royce C (1998). Efficacy and safety of CGP 56697 (artemether and benflumetol) compared with chloroquine to treat acute falciparum malaria in Tanzanian children aged 1–5 years. Trop Med Int Health.

[B21] Seidlein LV, Borjarg K, Jones P, Jaffar S, Pinder M, Grathman I, Royce C, McAdams K, Greenwood B (1998). A randomized controlled trial of artemether-benflumetol, a new antimalarial and sulfadoxine-pyrimethamine in the treatment of uncomplicated falciparum malaria in African children. Am J Trop Med Hyg.

[B22] Meremikwu M, Alaribe A, Ejemot R, Oyo-Ita A, Ekenjoku J, Nwachuckwu C, Ordu D, Ezedinachi E (2006). Artemether-lumefantrine versus artesunate plus amodiaquine for treating uncomplicated malaria in Nigeria: randomized controlled trial. Malar J.

[B23] Ashley EA, Stepniewska K, Lindegardh N, Anneberg A, Khan AM, Brockman AI, Singhasivanon P, White NJ, Nosten F (2007). How much fat is necessary to optimize lumefantrine oral bioavailability. Trop Med Int Health.

[B24] FMOH (2005). National Treatment guidelines for the treatment of malaria in Nigeria.

